# Metagenomic Functional Shifts to Plant Induced Environmental Changes

**DOI:** 10.3389/fmicb.2019.01682

**Published:** 2019-07-26

**Authors:** Svetlana N. Yurgel, Jacob T. Nearing, Gavin M. Douglas, Morgan G. I. Langille

**Affiliations:** ^1^Department of Plant, Food, and Environmental Sciences, Dalhousie University, Halifax, NS, Canada; ^2^Department of Microbiology and Immunology, Dalhousie University, Halifax, NS, Canada; ^3^Department of Pharmacology, Dalhousie University, Halifax, NS, Canada

**Keywords:** metagenome, functions, rhizosphere, tradeoff, network interaction

## Abstract

The *Vaccinium angustifolium* (wild blueberry) agricultural system involves transformation of the environment surrounding the plant to intensify plant propagation and to improve fruit yield, and therefore is an advantageous model to study the interaction between soil microorganisms and plant–host interactions. We studied this system to address the question of a trade-off between microbial adaptation to a plant-influenced environment and its general metabolic capabilities. We found that many basic metabolic functions were similarly represented in bulk soil and rhizosphere microbiomes overall. However, we identified a niche-specific difference in functions potentially beneficial for microbial survival in the rhizosphere but that might also reduce the ability of microbes to withstand stresses in bulk soils. These functions could provide the microbiome with additional capabilities to respond to environmental fluctuations in the rhizosphere triggered by changes in the composition of root exudates. Based on our analysis we hypothesize that the rhizosphere-specific pathways involved in xenobiotics biodegradation could provide the microbiome with functional flexibility to respond to plant stress status.

## Introduction

The rhizosphere is a hotspot of plant–microbiome interactions within soil environments. It is occupied by highly diverse microbial communities which are structurally and functionally influenced by plant and soil type (Philippot et al., 2013). Many previous studies of the metabolic capabilities of plant-associated microbiomes have identified specific functions linked to plant–microbiomes interaction, such as cell motility and root adhesion, metabolism of nitrogen, carbohydrates and vitamins, and xenobiotic degradation (Bulgarelli et al., 2015; Yan et al., 2017; Kamutando et al., 2018). However, it is not clear how these rhizosphere-specific (RS) functions are incorporated into the overall metabolic capabilities of soil microbiomes in general. In particular, it remains unclear whether microbial adaptation to the rhizosphere environment is associated with decreased general metabolic capabilities. The presence of this association would suggest that rhizosphere-associated microbes have some level of plant dependency whereby they rely on plant-produced compounds for their metabolism.

Herein, we conducted a co-occurrence analysis of the pathways found in bulk soil and rhizosphere metagenomes to identify niche-specific sub-networks and their interactions. We hypothesized that if there were a trade-off between rhizosphere and bulk soil metabolic capabilities then the pathway co-occurrence network would have a modular structure with strong positive interactions between niche-specific functions within each sub-network and negative interactions between rhizosphere and bulk soil-specific sub-networks. We were also interested in the identification of rhizosphere specific (RS) functional sub-networks that did not have strong negative interactions with bulk soil-specific (BSS) functional sub-networks, therefore representing an increment in functional repertoire of plant-associated microbiome.

*Vaccinium angustifolium* (wild blueberry) management is an attractive system to study plant–microbiome interaction since, in contrast to most agricultural systems that involve cultivation on plants in exogenous environments, this system involves transformation of the environments surrounding the plant to intensify plant propagation and to improve fruit yield (Hall et al., 1979; Eaton, 1988; Bell et al., 2009; Drummond et al., 2009). As a result the plant genotype and its native location become the only factors that remaining unchanged in wild blueberry managed habitats. In contrast, the management of the land, plant and soil affects land cover, wild blueberry fruit production and soil properties, which in turn can affect the diversity and structure of the bulk soil and plant-associated microbiome. Consequently, this system is an advantageous model to study the effects of environmental factors on soil and plant-associated microbiomes, the interaction between soil microorganisms and plant–host, and the functional differentiation of rhizosphere microbiomes.

In our previous studies (Yurgel et al., 2017, 2018) the application of both 16S and 18S rRNA amplicon sequencing provided us with some level of understanding of how deterministic factors, such as soil and plant properties shape the wild blueberry microbiome structure and define plant–microbiome interaction. We detected a significant effect of management on the structure of bacterial microbiome in the wild blueberry rhizosphere (Yurgel et al., 2017). Community correlation networks analysis identified several potential hub taxa with important roles in soil fertility and/or plant–microbe interaction and showed that bacterial and eukaryotic interactions became more complex along the soil-endosphere continuum, likely due to the increasing influence of host–plant on microbiome function (Yurgel et al., 2018).

However, the role of environmental factors in the functional assemblage of the wild blueberry microbiome has not been explored. Additionally, the indication that the structure of wild blueberry rhizosphere microbiome was affected by management raised a question of whether the observed taxonomic differences reflected niche-specific microbial functioning. For example, differences in the taxonomic composition of nematode-associated microbiomes did not reflect differences in their functional capabilities (Cheng et al., 2013), likely due to the substantial functional redundancy in microbial communities. Moreover, past work investigating the role of diversity in the selection of bacterial taxa and functions in soil and rhizosphere, suggested that functional traits were a key to the assembly of the rhizosphere microbiome (Yan et al., 2017). To explore this subject further we compared the functional profiles of rhizosphere microbiomes from managed and forest grown plants, which differ significantly in their taxonomic profiles (Yurgel et al., 2017). We were especially interested whether or not the taxonomic differences in rhizosphere microbiomes could be related to their functional capabilities.

## Materials and Methods

### Sample Collection and DNA Isolation

The wild blueberry root samples, rhizosphere and bulk soil used in this study were collected in August 2015 (Yurgel et al., 2017). The same DNA isolated from bulk and rhizosphere soils from Collingwood (45°36′53″ N 63°56′32″ W) and Debert (45°25′36″ N 63°29′47″ W) sampling sites (Supplementary Table S1) used for 16S and 18S rRNA amplicon analysis (Yurgel et al., 2017, 2018) was used for shotgun metagenome sequencing. The samples collection, processing and DNA isolation is described in Yurgel et al. (2017). In short bulk field soil samples were taken from the top 20 cm of the topsoil layer directly under blueberry plants in an X-shaped pattern with at least 30 m of distance between each collection point. The litter layer was removed and the samples were transported into the laboratory on ice, and immediately stored at −20°C for chemical characterization. After transportation to the lab, 5 g of each soil sample was sieved (2 mm) and immediately stored at −86°C until processing for DNA isolation. DNA was isolated from 0.25 g of the frozen filtered soil samples.

For the collection of rhizosphere samples wild blueberry rhizomes and associated roots were extracted from soil, vigorously shaken, placed in sterile bags, and transported to the laboratory on ice. Root samples were processed immediately after transportation to the laboratory. Blueberry roots were placed in a Falcon tube (50 ml) with 10% glycerol (40 ml) and vortexed until adhering soil had been visibly removed from the root. The roots were removed and the soil suspension was centrifuged at 3,000 × *g* for 15 min. The supernatant was decanted, and the soil pellets were transferred into 1.5-ml Eppendorf tubes and stored at −86°C. DNA was isolated from 0.25 g (wet weight) of each frozen rhizosphere soil sample.

### Shotgun Library Construction and Sequencing

One nanogram of DNA for each sample was subjected to Nextera XT (Illumina) library preparation. This was done as per the manufacturer’s instructions except the clean-up and normalization stages, which were completed using Just-a-Plate 96 PCR Purification and a Normalization Kit (Charm Biotech). Equal amounts of all barcoded samples were then pooled and sequenced in a shared 150+150 bp paired-end NextSeq run (Illumina High-Output v2 kit). The data generated for this study is available at the short read archive under BioProject PRJNA484230.

### Shotgun Metagenomics Pre-processing

The Microbiome Helper (Comeau et al., 2017) metagenomics workflow v2 was followed to process the shotgun metagenomics data with modifications to normalize functional abundance by average genome size within a sample. Briefly, raw paired-end reads were filtered using the KneadData v0.6.1 pipeline parallelized with GNU Parallel v20170322 (Tange, 2011). The filtering step included first trimming reads with Trimmomatic v0.36 (Bolger et al., 2014) in sliding windows of four nucleotides (nt) with a minimum quality score of 30. Trimmed sequences below 50 nt were removed. The next filtering step involved running Bowtie2 v 2.3.4.2 (Langmead and Salzberg, 2012) to remove contaminant reads that mapped human (hg38), PhiX, or *Vaccinium corymbosum* W8520 (Gupta et al., 2015) genomes with the options “—very-sensitive” and “–dovetail.” HUMAnN2 0.11.2 (Franzosa et al., 2018) was run on these filtered reads to identify the number of reads per kilobase (RPK) of UniRef50 gene families (Suzek et al., 2015) in each sample. UniRef50 gene families were then converted into KEGG orthologs using HUMAnN2’s built in mapping files. KEGG ortholog abundances were then normalized by the number of genome equivalents (average genome size/library size) found in each sample using Microbe Census (Nayfach and Pollard, 2015) to get reads per kilobase per genome equivalent (RPKG). Normalized KEGG orthologs were then mapped to KEGG pathways and KEGG modules using MinPath (Ye and Doak, 2009) and the PICRUSt2 script pathway_pipeline.py (Douglas et al., 2019) using the “–no_regroup” option and mapping files included with PICRUSt2 v2.1.4-b. MetaPhlAn2 v2.7.62 (Truong et al., 2015) was run with default options within HUMAnN2 to identify the relative abundances of taxa within each sample. A custom Python script was used to determine the GC-content of the microbiome overall and for the *V. corymbosum* reference genome.

HUMAnN2 tables with RPKG of metabolic pathways were transformed to BIOM tables and used for the generation of beta-diversity (weighted Bray–Curtis dissimilarity) (Bray and Curtis, 1957) metrics and analysis of variations in sample groupings explained by weighted Bray–Curtis beta-diversity dissimilarity (Adonis tests, 999 permutations) using QIIME wrapper scripts (Caporaso et al., 2010). An important assumption of this approach is that the read depth per sample is sufficiently high to accurately estimate the abundance of the gene families underlying these metabolic pathways. Analysis of taxonomic and functional profiles was performed using the STAMP software package (Parks et al., 2014). Corrected *p*-values (*q*-values) were calculated based on Benjamini–Hochberg FDR multiple-test correction. Features with (Welch’s *t*-test) *q*-value < 0.05 were considered significant and were thus retained.

### Co-occurrence Network Construction and Analysis

The co-occurrence analysis was performed using the CCREPE (Compositionality Corrected by REnormalization and PErmutation) R package (Schwager and Huttenhower, 2016, Unpublished) with 1000 bootstrap iterations and default setting. This package has previously been used to construct co-occurrence networks from microbial sequencing data (Vazquez-Baeza et al., 2016; Yurgel et al., 2018). This network uses a novel similarity measure, the N-dimensional checkerboard score (NC-score) (Stone and Roberts, 1990), which is particularly appropriate to compositions derived from microbial community sequencing data. First, the co-occurrence and co-exclusion patterns in the samples were scored. The results were filtered to remove non-statistically significant relationships. We generated the network based on strong correlations with *p*-values < 0.001. The networks were visualized with Cytoscape (Shannon et al., 2003) and were represented as graphs with microbial functions as vertices/nodes and the edges as interaction types.

## Results

### Sequencing and Data Processing

A total of 44,641,544, 70,129,920, 19,394,324, and 29,124,986 raw reads were obtained from 8 field bulk, 8 field rhizosphere, 4 forest bulk, and 3 forest rhizosphere soil samples, respectively. After assembly, quality control filtering and removal of artificial sequences produced by sequencing artifacts and plant sequences 9,412,563, 9,245,366, 2,864,804, and 3,756,327 high-quality reads were retained in field bulk soil, field rhizosphere, forest bulk, and forest rhizosphere metagenomes respectively (Supplementary Table S2). Interestingly, forest rhizosphere metagenomes exhibited distinct genome properties. It had a significantly lower (Tukey’s test *p* < 0.05) mean guanine and cytosine (GC) value (∼52%) compared to field rhizosphere (∼57%) and both field (∼59%) and forest (59%) bulk metagenomes.

### Taxonomic Composition of Bulk Soil and Rhizosphere Metagenomes

Using a unique clade-specific marker gene approach (Truong et al., 2015) we identified a total of 22 bacterial taxa across all samples. *Proteobacteria*, *Acidobacteria*, and *Actinobacteria* were the predominant bacterial phyla in both bulk soil and rhizosphere samples. A similar taxonomic profile of soil microbiomes obtained by shotgun metagenome analysis was previously reported (Fonseca et al., 2018; Kamutando et al., 2018). A versatile soil bacteria capable of carbon and nitrogen fixation, *Rhodopseudomonas palustris*, was the most abundant bacterial species represented by >14% of all high-quality reads (Supplementary Table S3) and *Rhodococcus erythropolis* capable of degrading, toluene, naphthalene, herbicides, and other environmental pollutants (Curragh et al., 1994), was represented by ∼1% of all high-quality reads. We also identified *Pseudomonas stutzeri* (Parte and Kharat, 2019) a bacterial species capable of denitrifying and potentially degrading insecticides, as well as the nitrogen fixer *Bradyrhizobium japonicum*, and *Granulicella mallensis* (Rawat et al., 2012) a versatile heterotroph capable of degrading plant-based carbon polymers (Supplementary Table S3). Interestingly, the human pathogen *Mycobacterium intracellulare* was also identified, which corroborates confirming previous reports suggesting soil as its potential environmental reservoir (Wallace et al., 2013; Honda et al., 2016).

### Functional Characteristics of Bulk and Rhizosphere Microbiomes

In total, 5321 KEGG orthologs (KOs) comprising 200 KEGG pathways, 304 KEGG modules and 40 KEGG categories were identified in the study. Among them amino acid metabolism, carbohydrate metabolism, metabolism of cofactors and vitamins, metabolism of terpenoids and polyketides, lipid metabolism, and xenobiotics biodegradation and metabolism were the top KEGG functional categories identified representing 21, 14, 10, 9, 8 and 7% of all high-quality reads, respectively. The top 18 functional categories are shown in Supplementary Figure S1.

An analysis of the strength and statistical significance of sample groupings (Adonis test) indicated that niche (bulk soil and rhizosphere) environments were associated with differences in their microbiome’s functional composition. We found differences in the functional characteristics of each microbiome at the KEGG modules, pathways and KO levels (Table 1). When the microbiomes from managed and natural habitats were analyzed together by variations in sample groupings (Adonis test) explained by weighted Bray–Curtis beta-diversity dissimilarities, ∼20% (*R*^2^ = 0.201, *p* < 0.001), 17% (*R*^2^ = 0.174, *p* < 0.001), and 17% (*R*^2^ = 0.17460, *p* < 0.05) of functional variations between bulk soil and rhizosphere communities were detected for the modules, pathways and KO levels respectively. More specifically 51 modules were in differential relative abundance between bulk soil and rhizosphere samples. A number of sugar biosynthetic modules as well as fatty acid and amino acid biosynthetic modules were enriched in bulk soils compared to rhizosphere samples. On the other hand the rhizosphere samples were enriched for amino acid and sugar transport system modules. The 10 most relatively abundant modules overrepresented in bulk soil or in rhizosphere are shown in Supplementary Figure S2.

**TABLE 1:** Variation in sample groupings as explained by Bray–Curtis dissimilarity.

**KEGG hierarchy/sample grouping**	**Modules**	**Pathways**	**KOs**
Bulk soil vs. rhizosphere	0.201^∗∗∗^	0.174^∗∗∗^	0.160^∗^
Field bulk soil vs. field rhizosphere	0.230^∗∗^	0.196^∗∗^	0.185^∗∗^
Forest bulk soil vs. forest rhizosphere	0.310^∗^	0.283	0.275^∗^
Field rhizosphere vs. forest rhizosphere	0.161	0.131	0.137
Field bulk soil vs. forest bulk soil	0.083	0.072	0.100

*HUMAnN2 module, pathway and KO RPKG tables were used with the QIIME pipeline for Adonis tests to assess whether their Bray–Curtis dissimilarity were related to sample groupings by niche, 999 permutations, *R*^2^, ^∗^*p* < 0.05, ^∗∗^*p* < 0.01, ^∗∗∗^*p* < 0.001.*

**FIGURE 1:**
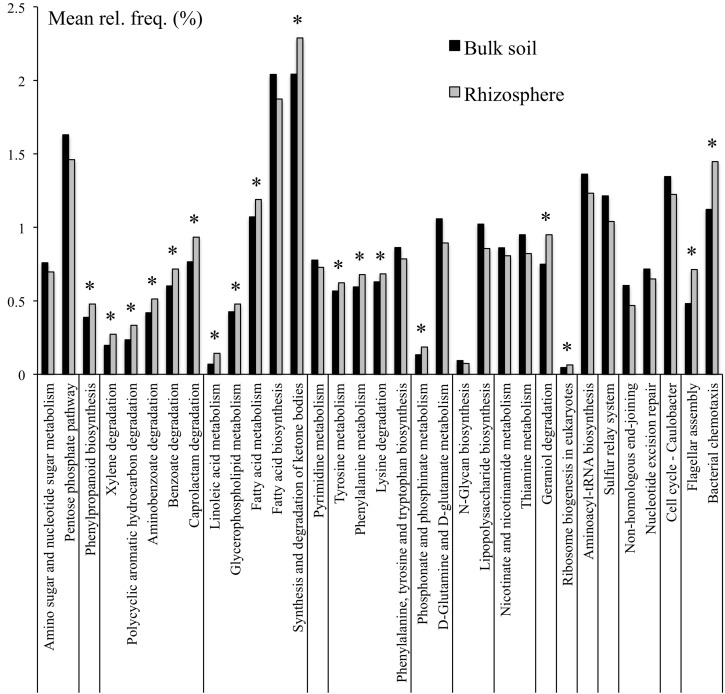
KEGG pathways that were at differential relative abanances between bulk soil and rhizosphere samples. Only pathways with mean relative frequencies > 0.5% are shown. Corrected *p*-values (*q*-values) were calculated based on Benjamini–Hochberg FDR multiple test correction. Features with (Welch’s *t*-test) *q*-value < 0.05 were considered significant and were thus retained. ^*^Pathways overrepresented in rhizosphere.

To further refine the functional potential of the bulk soil and rhizosphere microbiomes, the relative abandances of pathways were compared. In total 35 pathways were differentially represented between bulk soil and rhizosphere (Supplementary Table S4). In particular, the pathways constituting carbohydrate, glycan, nucleotide, and vitamin metabolism, as well as folding, sorting and degradation, and replication and repair were overrepresented in the bulk soil compared to rhizosphere samples. In contrast, the pathways involved in degradation of lysine, xenobiotics and plant-derived terpenoids, as well as tyrosine and phenylalanine metabolism and bacterial chemotaxis and flagellar assembly were overrepresented within the field rhizosphere samples (Figure 1).

In total 427 KOs were differentially enriched in field bulk soils and rhizosphere. The 15 most abundant KOs overrepresented within each soil-types are shown in Supplementary Figure S3. The rhizosphere-specific (RS) KOs included K01075, which is found in pathways involved in the biosynthesis of secondary metabolites and benzoate degradation, and K04073, which is found in the pathways involved in the degradation of butanoate, benzoate, xylene, dioxin, and other aromatic compounds. The putrescine transport system permease protein, K11074, and arginine decarboxylase K01584, which is involved in arginine and proline metabolism were also overrepresented in rhizosphere samples. The putative ABC transport system ATP-binding protein, K02003, and the cold shock protein (beta-ribbon, CspA family), K03704 were the most abundant bulk-soil-specific (BSS) KOs.

#### Managed vs. Natural Habitats

The analysis of strength and statistical significance of sample groupings by management indicated that this parameter did not significantly influence the functional characteristics of rhizosphere and bulk soil microbiomes at the modules, pathways, or KO levels (Table 1). We also did not identify any microbiome functions at any functional level that were differently represented between the rhizosphere of managed and unmanaged plants or between the bulk soils from managed and unmanaged plant habitats.

### Functional Network Interaction

**FIGURE 2:**
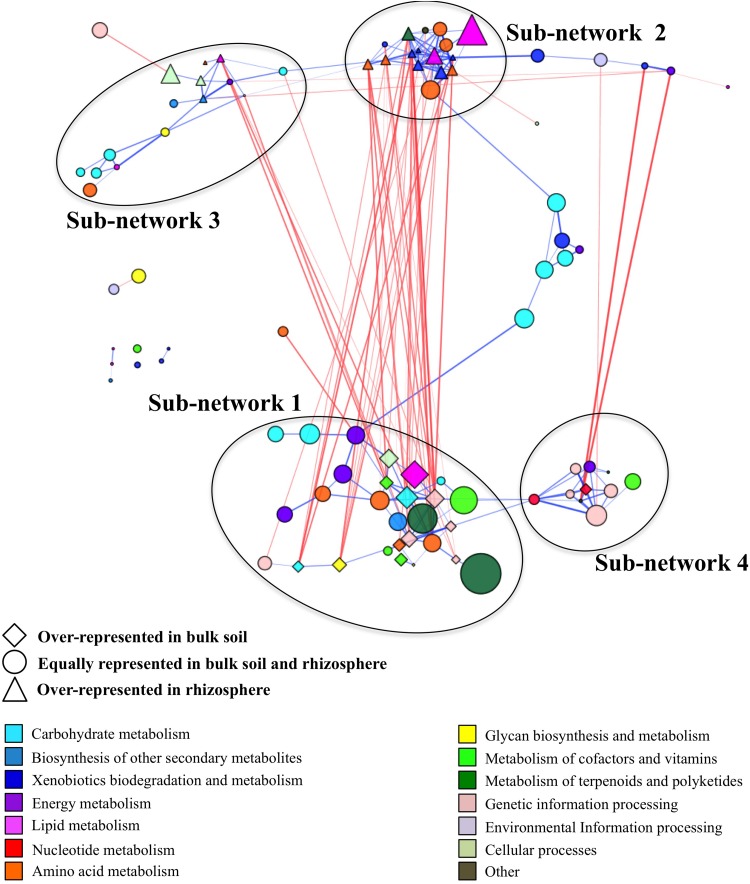
Co-occurrence network generated based on pathway relatively abundances in term of reads per kilobase per genome equivalent (RPKG) within bulk soil and rhizosphere samples. The size of the node is proportional to each pathway’s relative abundance across all samples. The lines (i.e., edges) connecting nodes represent a co-occurrence relationship that can be either positive (blue) or negative (red). The intensity of the color and the length of the edges represent the strength of relationship. The positions of the nodes within modules were manually adjusted for better visualization.

We generated a co-occurrence network by correlating the relative abundances between pathways identified in samples from the bulk field and forest soil and the rhizosphere of managed and unmanaged plants. The final co-occurrence network contained 92 pathways forming 49 negative and 180 positive correlations (Supplementary Table S5). This co-occurrence network incorporated the pathways represented by ∼73% of all high-quality reads obtained in the study (Supplementary Table S6). We used the “edge-weighted spring embedded” layout to visualize this interaction. This layout allowed us to visually identify functional clusters by pulling positive correlations (blue) together and pushing negative correlations (red) apart (Figure 2). The functional co-occurrence network was modular and contained four well-defined sub-networks. Two of these sub-networks, Sub-networks 1 and 2, had strong negative correlation with each other, while two others, Sub-networks 3 and 4, were loosely interconnected within the network.

#### Sub-Network 1

It contained 28 pathways with 13 pathways overrepresented in bulk soil (Supplementary Table S6). It had 39 negative interactions with outside nodes and 55 positive interactions within the sub-network with average positive and negative interaction scores of 0.62 and −0.63, respectively. This sub-network was the largest sub-network in the co-occurrence network and it encompassed ∼32% of total high-quality reads identified in the entire microbiome. It contained major functional categories essential for microbiome metabolism and function. The most relatively abundant functional categories found in this sub-network included the metabolism of terpenoids and polyketides (biosynthesis of antimicrobials vancomycin and ansamycins), carbohydrate metabolism, genetic information processing, metabolism of cofactors and vitamins, environmental information processing and amino acid metabolism (Table 2). Since nearly half of the pathways in Sub-network 1 were significantly overrepresented in bulk soil, this sub-network was considered BSS. Many pathways from this sub-network formed strong negative correlations with pathways from Sub-network 2 producing 32 negative interactions.

**TABLE 2:** Distribution of KEGG functional categories in the pathway co-occurrence network.

**KEGG functional categories**	**Mean relative frequency (%)**				
	**Sub-network 1**	**Sub-network 2**	**Sub-network 3**	**Sub-network 4**	N/M^*^
Carbohydrate metabolism	5.2	0	2.4	0	2.4
Energy metabolism	3.5	0	0.3	0.7	0.3
Lipid metabolism	2	3.3	0.7	0	0.2
Nucleotide metabolism	0	0	0	1.4	0
Amino acid metabolism	3.3	4.9	1	0	1.7
Glycan biosynthesis and metabolism	0.1	0	0.5	0	0.90
Metabolism of cofactors and vitamins	4.2	0	0	1	0.5
Metabolism of terpenoids and polyketides	5.9	1	0	0.1	0
Biosynthesis of other secondary metabolites	1.2	0	0.9	0	0.1
Xenobiotics biodegradation and metabolism	0	3.6	0	0	2.0
Genetic information processing	4.5	0	0	3.5	1
Environmental information processing	3.6	0	0	0.1	0.6
Cellular processes	1.3	0	1.9	0	0.1

^*^Not associated with sub-networks.

#### Sub-Network 2

It had 33 negative and 66 positive interactions with average positive and negative interaction scores of 0.62 and −0.62, respectively. It was represented by 17 pathways containing ∼13% of total high-quality reads and was considered as RS since 11 pathways found in this sub-network were significantly overrepresented in rhizosphere (Supplementary Table S6). Sub-network 2 contained a smaller subset of functional categories, some of which might be involved in microbiome responses to environmental factors. For example, the functional category xenobiotics biodegradation was one of the most relatively abundant functional categories in Sub-network 2 and was overrepresented in the sub-network compared to all other sub-networks (Table 2). Additionally, the functional category amino acid metabolism, comprising amino acid degradation and the metabolism of phenolic amino acids, was the most relatively abundant functional category in Sub-network 2. Geraniol degradation was also a part of this sub-network.

**FIGURE 3:**
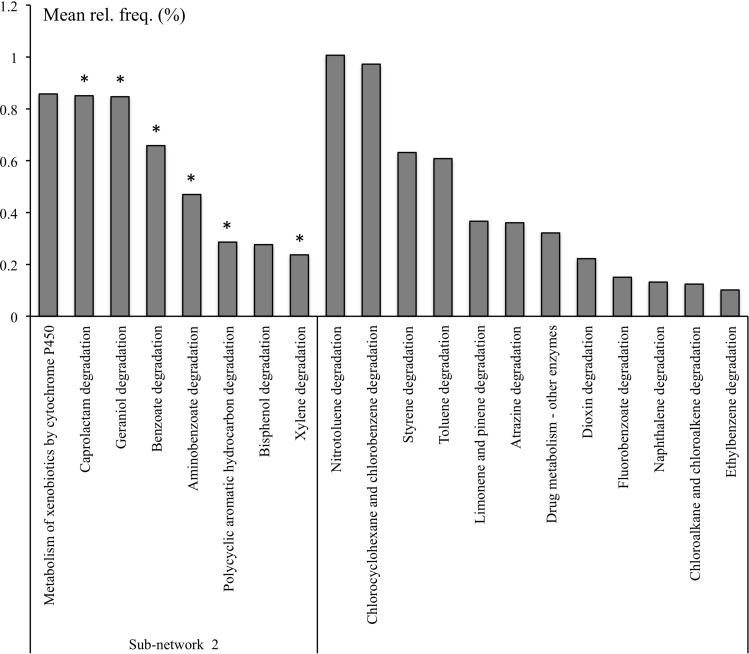
Selected KEGG pathways of xenobiotics, terpenoids, and polyketides metabolism in microbial metagenomes from bulk and rhizosphere soils from natural and managed habitats. ^*^Pathways overrepresented in rhizosphere.

The pathways involved in the degradation of aminobenzoate, caprolactam, benzoate, xylene, polycyclic aromatic hydrocarbon (PAH) were significantly overrepresented in the rhizosphere microbiome and were a part of RS Sub-network 2 identified in the co-occurrence network (Figure 3). Bisphenol degradation and the metabolism of xenobiotics by cytochrome P450 were also part of the Sub-network 2 but they could not be attributed to RS pathways by statistical analysis (Supplementary Table S4). However these pathways might be considered as a part of RS functions considering their significant association with several niche-specific pathways. For example, bisphenol degradation (ko00363) was positively correlated with RS metabolism of phenylalanine (ko00360) and aminobenzoate degradation (ko00627), and the metabolism of xenobiotics by cytochrome P450 (ko00980) was positively correlated with RS PAH degradation (ko00624) (Figure 3 and Supplementary Table S3). With the exception of the metabolism of xenobiotics by cytochrome P450 and degradation of bisphenol and PAH, which were represented with 36, 42, and 35% of KOs, respectively, the other pathways involved in xenobiotics, terpenoids, and polyketides metabolism found in Sub-network 2 and were represented with at least 68% of KOs (Supplementary Table S7).

#### Sub-Network 3

It had 9 negative and 27 positive interactions with average positive and negative interaction scores of 0.61 and −0.62, respectively. It was represented by ∼7.8% of total high-quality reads obtained in the study and contained 15 pathways from KEGG functional categories. Five pathways belonging to this Sub-network, phenylpropanoid biosynthesis, glycerophospholipid, phosphonate and phosphinate metabolism, bacterial samples (Table 2 and Supplementary Table S6). However, the rest of the pathways found in the Sub-network 3 were similarly represented in bulk soil and the rhizosphere suggesting a boarder role of this sub-networks in both plant-associate and free-living soil microbiome function.

#### Sub-Network 4

It had 3 negative and 23 positive interactions with average positive and negative interaction scores of 0.64 and −0.81, respectively and contained 11 pathways comprised by ∼7.8% of total high-quality reads (Supplementary Table S6). Genetic information processing and nucleotide metabolism were the most relatively abundant categories in the sub-network (Figure 2). With the exception of pyrimidine metabolism all other pathways comprising Sub-network 4 were similarly represented in bulk and rhizosphere soils suggesting important role of these functional capabilities in both bulk soil and rhizosphere microbiomes.

## Discussion

In our previous work we used amplicon-based approaches to study microbial communities associated with wild blueberry production system (Yurgel et al., 2017, 2018). This enabled the investigation of how environmental and plant factors affect microbial community structure, provided an integrative view on inter-kingdom interactions in soil and plant associated microbiomes, and identified microbial taxa with potential importance in plant health and production. However, until now the functional potential of the wild blueberry soil microbiome remained largely unknown. In this study we applied shotgun metagenome sequencing to study the functional characteristics of these communities.

As previously reported (Tessler et al., 2017), whole-metagenome sequencing coupled with clade-based taxonomic algorithm (MetaPhlAn2) approach for the taxonomic profiling of microbial communities was far less comprehensive compared to rRNA gene sequencing analysis. For example, only 22 microbial taxa were identified in our study compared to 996 eukaryotic and 6,802 bacterial OTUs identified by 18S and 16S rRNA sequencing (Yurgel et al., 2018). However, in agreement with our previous reports *Proteobacteria*, *Acidobacteria*, and *Actinobacteria* were the predominant bacterial phyla identified in the wild blueberry-associated soil microbiome (Yurgel et al., 2017, 2018).

### Nucleotide Composition

Interestingly, GC-content varied greatly between the forest and field rhizosphere metagenomes. It was previously reported that both phylogeny and the environment affected nucleotide composition of microbial communities from diverse ecological niches (Reichenberger et al., 2015). In our study, the difference in GC-content was only detected between field bulk soil and rhizosphere of managed plants. On the other hand, both plant-proximity and management affected the taxonomic composition of wild blueberry microbial communities (Yurgel et al., 2017, 2018) confirming the importance of environments in shaping microbial nucleotide composition.

It is also important to notice that the raw reads obtained from rhizosphere and bulk soil niches also exhibited distinct GC-signature. Both field and forest bulk soil metagenome had significantly higher (*p* < 0.05) GC-content (∼60%) compared to rhizosphere metagenomes (∼50%). We calculated that GC-content of *V. corymbosum* scaffold was ∼38%. Therefore, our initial hypothesis was that residual plant tissue contributed to rhizosphere metagenomes decreasing their GC-content. In our analysis we filtered raw reads against genome of *V. corymbosum* (Bian et al., 2014) to remove plant derived reads. However, after quality control filtering and removal of contaminant sequences forest rhizosphere metagenome still exhibited lower GC-content compared to the other metagenomes, suggesting that the presence of plant DNA in the raw metagenome was not the only reason for the distinct GC-signature of rhizosphere metagenome.

### Carbon-Nitrogen Cycling

Microorganisms play a critical role in carbon-nitrogen biogeochemical cycling. In agreement with this, carbohydrate and nitrogen metabolism were the most abundant KEGG functional categories identified in our study. A purple photosynthetic bacterium from the *Bradyrhizobiaceae* family, *Rhodopseudomonas palustris*, was the most abundant bacterial species in our samples and is known to have extraordinary metabolic capabilities. It can acquire carbon by catabolism of organic molecules and carbon fixation, use light and organic and inorganic compounds for energy, as well as fix atmospheric di-nitrogen, actively participating in most steps of carbon and nitrogen cycle (Larimer et al., 2004). Several other bacterial taxa involved in nitrogen and carbon cycling from the *Bradyrhizobiaceae*, *Beijerinckiaceae*, *Burkholderiaceae*, and *Rhodopseudomonas* families were identified in our study. This relatively high abundance of bacteria with potential nitrogen and carbon fixation capabilities might be attributed to the adaptation of the microbiome to low fertility soils typical of wild blueberry habitats (Yurgel et al., 2017). The denitrifying bacteria *Pseudomonas stutzeri* (Lalucat et al., 2006) was also detected in the microbiome. Although the relative abundance of this bacterium was much lower compared to potential nitrogen fixers, probably because of slow rate of nitrification in acidic soils.

### Niche-Specific Functions

The functional characteristics varied significantly between rhizosphere and bulk soil microbiomes. Depending on the level of functional annotation (modules, pathways, or KOs), between 16 and 23% of the variation in community function was niche specific. Carbohydrate metabolism was underrepresented, while the transport of sugars and by product of amino acid degradation, putrescine, were overrepresented in the rhizosphere, potentially due to compensation for decrease in some metabolic capacity of the plant-associated microbiome. Additionally, the degradation of complex organic compounds was overrepresented in the rhizosphere. Previously we showed that the community structure was significantly influenced by the plant, with between 10 and 14% of bacterial community variation being niche-specific (Yurgel et al., 2017, 2018), suggesting that some functional changes between bulk soil and rhizosphere could be attributed to the alteration in microbial community structure.

### Functional Stability of Bulk Soil Microbiome

Management was a significant factor influencing the diversity and structure of bulk soil microbiome with 12% of bacterial community variation in bulk soil being attributed to management (Yurgel et al., 2017). However, no significant effect of the management on the bulk soil microbiome function was detected in our study. Furthermore, the functional annotation of bulk soil microbiomes from managed and natural habitats were very similar and no significant differences in the mean relative frequency of functions were detected between them. This data suggested a stronger functional stability of soil microbiome compared to the community structure. Similar functional stability of nematode-associated microbiome was reported previously (Cheng et al., 2013).

### Functional Co-occurrence Network Interaction

To extend our view on the functional capabilities within the microbiome the interaction between metabolic pathways was investigated using a co-occurrence network analysis. This approach allowed us to extend the set of niche-specific pathways providing more comprehensive insight on overall microbiome metabolism and its functional specialization. We also performed statistical analysis of differences in the mean relative frequency of pathways between combined rhizosphere and bulk soils samples. As expected, in addition to 35 pathways, which were differentially represented in bulk soil and rhizosphere metagenomes, 14 additional pathways were identified as niche-specific by association. For example, bisphenol degradation was included into RS pathways category by its strong co-occurrence association with phenylalanine and aminobenzoate degradation, which were significantly overrepresented in rhizosphere metagenome.

### Xenobiotics Biodegradation and Metabolism

The potential of microbial communities to cycle natural halogens and aromatic compounds in soils is well-documented (Curragh et al., 1994; Weigold et al., 2016; Hlihor et al., 2017; Vergani et al., 2017; Hussain et al., 2018). In agreement with these previous findings, xenobiotics biodegradation and metabolism was among the most relatively abundant functional categories annotated in the metagenome, represented by 7% of all high-quality reads. All but three pathways belonging to this category were identified in our study including degradation of haloalkanes, herbicide atrazine, and aromatic compounds, such as bisphenol, naphthalene, chlorocyclohexane, chlorobenzene, nitrotoluene, styrene, caprolactam, toluene, benzoate, aminobenzoate, PAHs, fluorobenzoate, and dioxin. Herbicide atrazin was traditionally used in management of perennial grasses in wild blueberry fields, but was banned for use in 2013. The samples used in the study were collected in summer 2015, which indicates the long lasting effect of this compound on microbial functional potential.

Around 46% of all high-quality reads annotated as xenobiotics biodegradation and metabolism category comprised pathways which were either significantly overrepresented in rhizosphere or were strongly associated with RS pathways. The enrichment of the pathways involved in degradation of complex organic compounds in the rhizosphere could be attributed to several possible mechanisms. These mechanisms include: an increase in plant-derived complex molecules with the proximity to the plant, the stimulation of microbial biodegradation of organic pollutants by plant rhizo-deposits and inducers, and root exudate facilitated co-metabolic degradation (Haby and Crowley, 1996; Shaw and Burns, 2003; Grandy and Neff, 2008; Toyama et al., 2013).

### Overall Functional Structure of Wild Blueberry Microbiome

The pathway co-occurrence network contained four well-defined sub-networks. Sub-network 1 was considered BSS, Sub-network 2 was considered RS, and Sub-networks 3 and 4 was considered niche-independent. The Sub-network 2 comprised many pathways involved in xenobiotics biodegradation and metabolism of terpenoids and polyketides. Many of these functions were negatively correlated with the basic metabolic pathways from Sub-network 1. 32 negative correlations were detected between the sub-networks, giving on average ∼1.9 negative correlation per node in Sub-network 2. Taking into consideration that the majority of pathways in Sub-network 1 were essential for general microbiome function, we can conclude that functions in Sub-network 1 and 2 formed a single unit, where the gain of the specific functions essential for adaptation of the microbiome to the rhizosphere environment were linked to the loss of some basic metabolic functions. For example, glycolysis/gluconeogenesis, pentose phosphate pathway, TCA cycle, carbon fixation, metabolism of cofactors and vitamins and genetic information processing were underrepresented in the rhizosphere. This shift in genome functions might be beneficial for the survival of the microbes in the rhizosphere but might also reduce the ability of these microbes to withstand stresses in bulk soils.

Many basic metabolic functions were similarly represented in bulk soil and rhizosphere. For example, within the carbohydrate metabolism category, starch, sucrose, pyruvate, galactose, ascorbate, glyoxylate, dicarboxylate, aldarate, propanoate, butanoate, and C_5_-branched dibasic acid metabolism and pentose and glucuronate interconversions were not niche specific, indicating functional capability of both in bulk soils and rhizosphere microbiome to metabolize a divers set of sugars and organic acids. In general, primarily metabolites (sugars, organic acids, and amino acids) are predominant compounds found in root exudates, while secondary metabolites (polycyclic aromatic compounds and phytohormones) are less abundant (van Dam and Bouwmeester, 2016). However, the composition of root exudates is affected by plant genotype, age, and biotic and abiotic factors (Chaparro et al., 2013; Mhlongo et al., 2018). It was proposed that plant root exudates are derived from the most abundant metabolites available at the time. For example, even small changes in plant water status can impact the composition of root exudates. It was shown that *Quercus ilex* (holm oak) root exudates contained 71% of secondary metabolites and 81% of primary metabolites under drought stress and the recovery phase, respectively (Gargallo-Garriga et al., 2018). The functional structure of the wild blueberry soil microbiome indicated that the basic metabolic functions necessary for both bulk soil and rhizosphere microbiomes are complemented with rhizosphere-specific functions, which could provide the microbiome with additional functional capabilities to respond to environmental fluctuations in rhizosphere triggered by changes in the composition of root exudates. Interestingly, the majority of the pathways from Sub-network 2 were involved in the degradation of secondary metabolites, such as polycyclic aromatic compounds and terpenoids suggesting the importance of these pathways in microbiome responses to plant stress status.

On the other hand, Sub-networks 3 and 4 were less connected in the network. We hypothesize that this low integration of the pathways comprising Sub-network 3 into general microbiome metabolic functions (Sub-networks 1 and 2) could provide the microbiome with additional functional capabilities for interaction with host–plant, including chemotaxis, plant–pathogen interaction, and lipid and glycan metabolism.

## Conclusion

We investigated the functional characteristics of the wild blueberry soil microbiome with shotgun metagenomics sequencing. The metagenome GC-content varied greatly between rhizosphere microbiomes from managed and unmanaged habitats indicating the importance of environments in shaping microbial nucleotide composition. A relatively high abundances of microorganisms with potential nitrogen and carbon fixation capabilities were detected suggesting an adaptation of the microbiome to low fertility soils typical for wild blueberry habitats. In contrast to the rhizosphere metagenome, the bulk soil metagenome exhibited functional stability indicating similar functional repertoire in microbiomes associated with soils from managed and natural habitats. Lower relative abundance in general metabolic functions, such as carbohydrate and amino acid metabolism and higher relative abundance in sugar and putrescine transport, as well as degradation of complex organic compounds were detected in the rhizosphere microbiome. The analysis of the pathway co-occurrence network extended the set of niche-specific functions and provided a better understanding of overall microbiome metabolism and its functional specialization. The strong modular structure of metagenome indicated a potential trade-off between functional adaptations of microorganisms to the rhizosphere environment and its basic metabolic function. Based on our analysis we hypothesize that the rhizosphere-specific pathways involved in biodegradation xenobiotics and terpenoids could provide the microbiome with functional flexibility to respond to plant stress status.

## Data Availability

Publicly available datasets were analyzed in this study. This data can be found here: https://www.ncbi.nlm.nih.gov/bioproject/?term=PRJNA484230.

## Author Contributions

SY and ML obtained funding and analyzed and discussed the results. SY designed the study, performed data pre-possessing, and wrote the manuscript. SY and JN performed the bioinformatics analyses. GD assisted with the bioinformatics analyses. SY, JN, GD, and ML participated in the production and the final version of the manuscript.

## Conflict of Interest Statement

The authors declare that the research was conducted in the absence of any commercial or financial relationships that could be construed as a potential conflict of interest.
