# Supplementary figures and images for: Metagenomic Functional Shifts to Plant Induced Environmental Changes
Source: Front Microbiol. 2019 Jul 26;10:1682. doi: 10.3389/fmicb.2019.01682 (PMC6676915; doi:10.3389/fmicb.2019.01682)

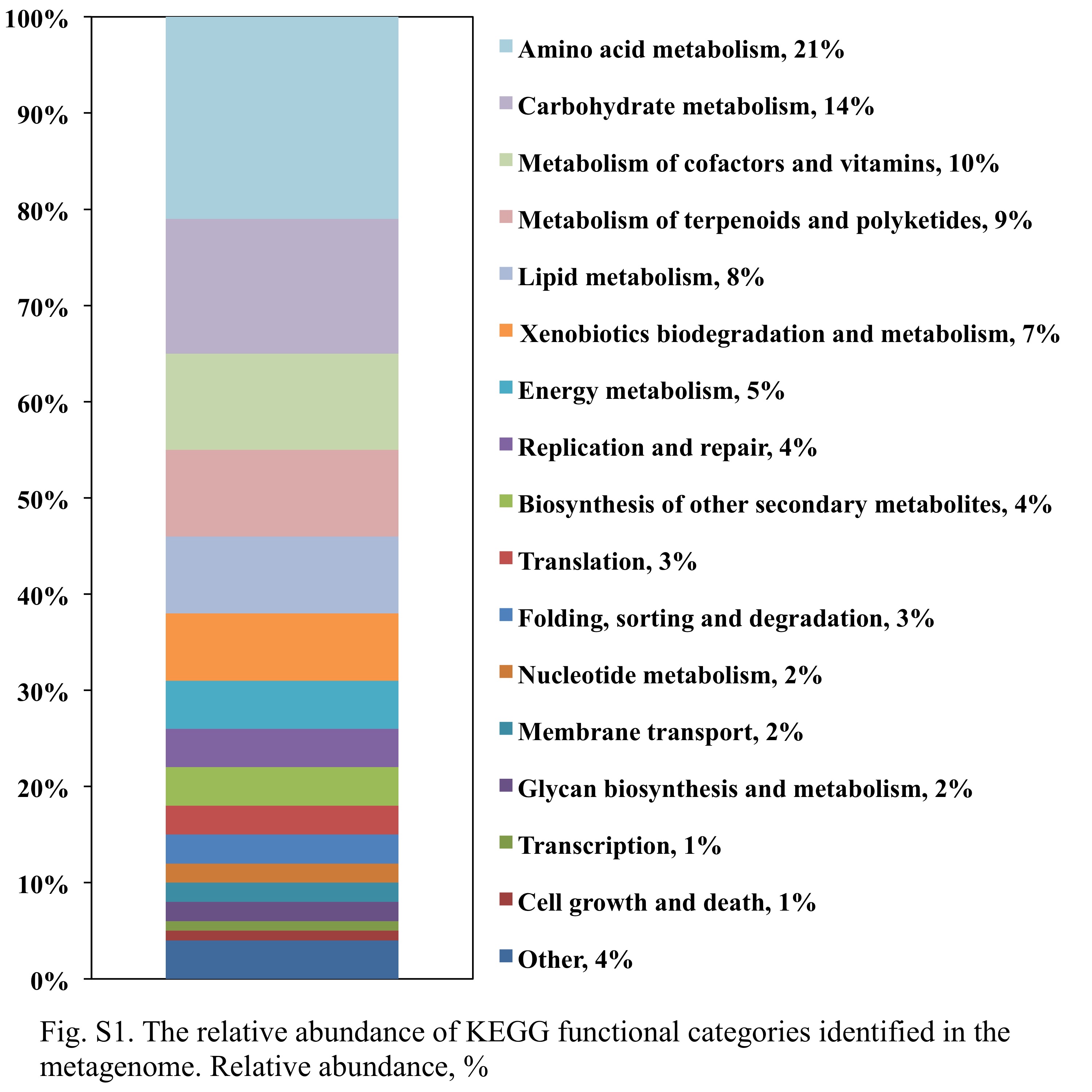

Supplement: Supplementary file 1 [file Image_1.JPEG]

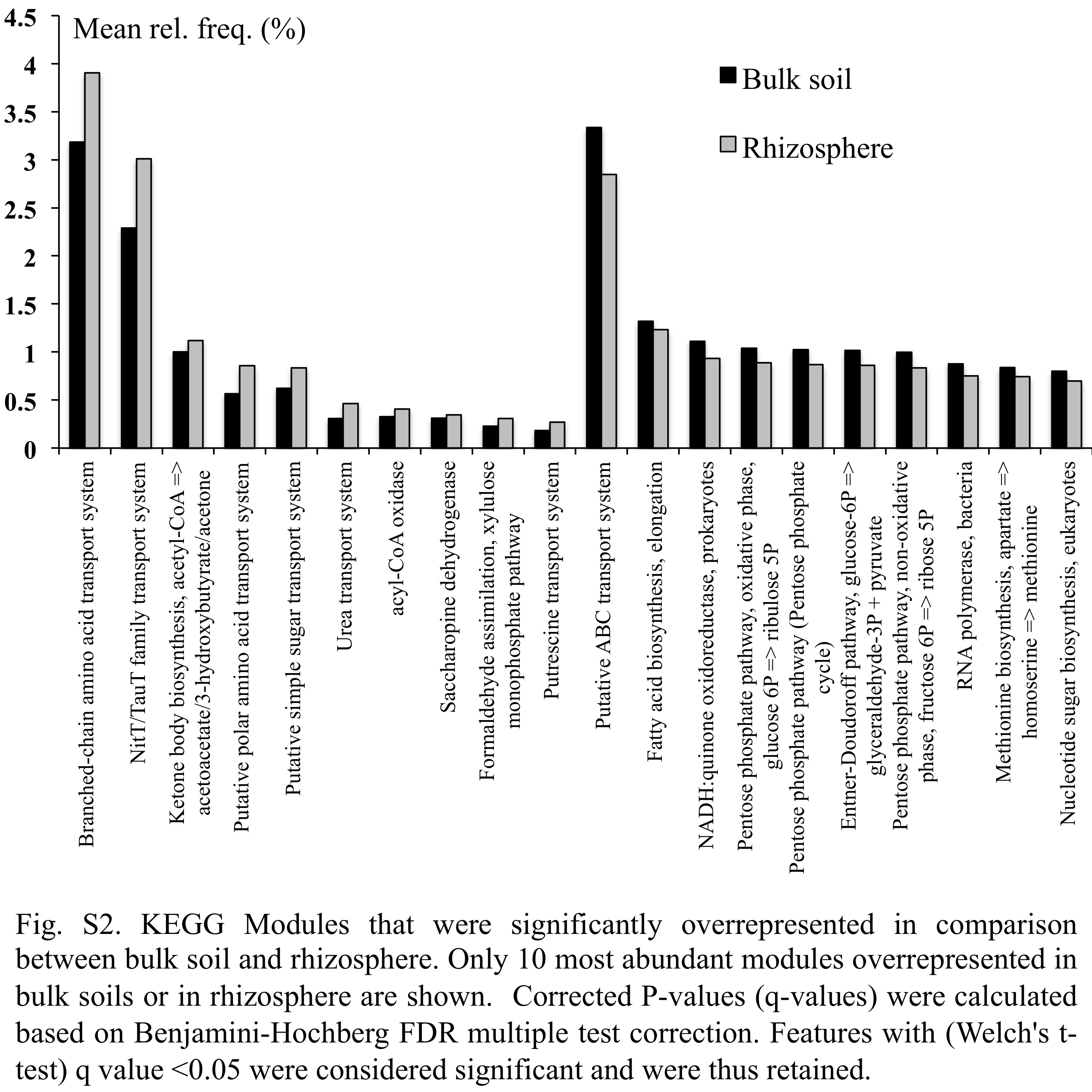

Supplement: Supplementary file 2 [file Image_2.JPEG]

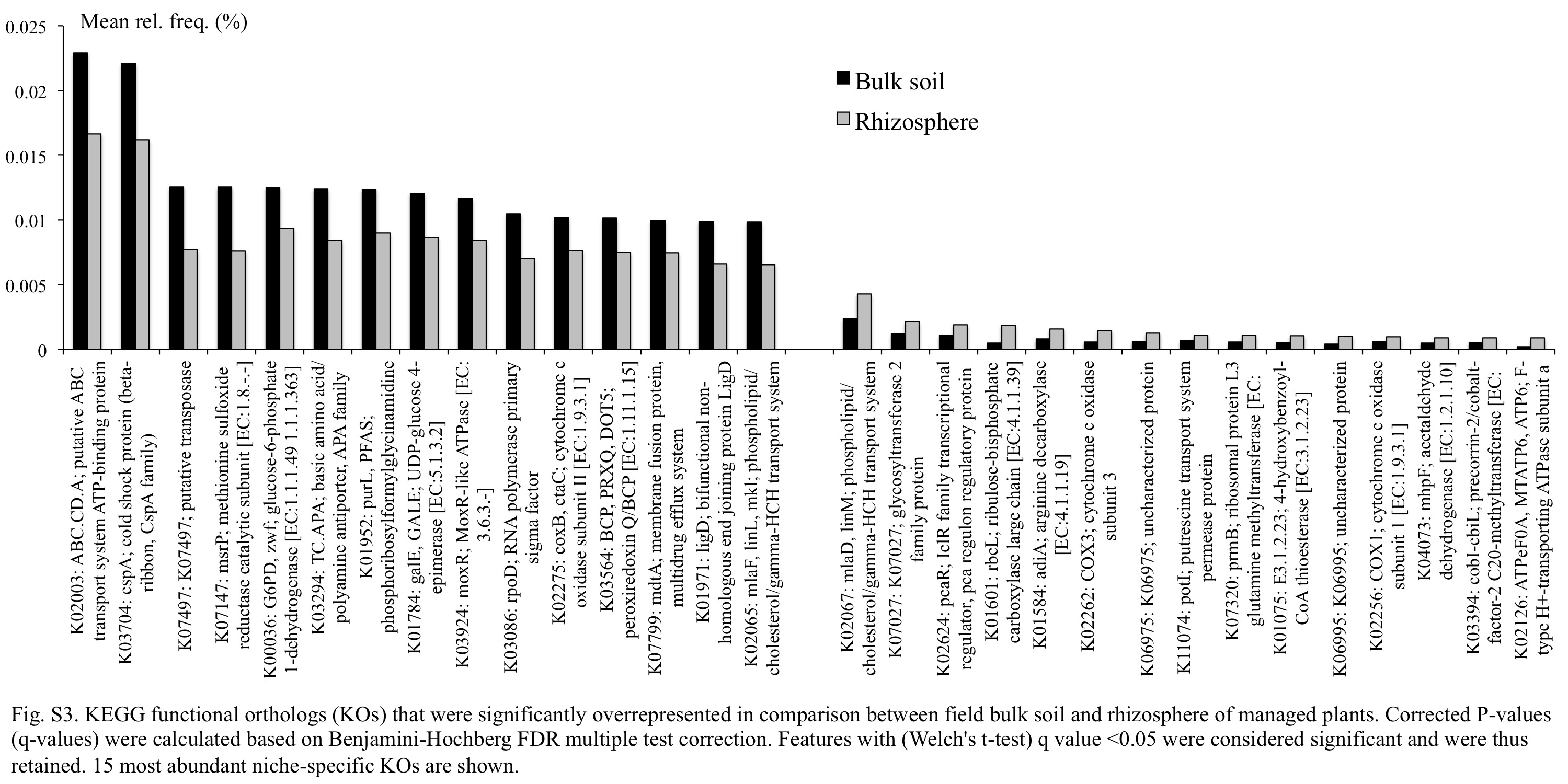

Supplement: Supplementary file 3 [file Image_3.JPEG]
