# Supplementary material for: Metagenomic Functional Shifts to Plant Induced Environmental Changes
Source: Front Microbiol. 2019 Jul 26;10:1682. doi: 10.3389/fmicb.2019.01682 (PMC6676915; doi:10.3389/fmicb.2019.01682)
Supplement: Supplementary file 4 [file Table_1.DOCX]

Table S1. Description of samples

| Sample type | Location | Number of samples |
| --- | --- | --- |
| Bulk soil within close proximity to roots of blueberry plants from forest, Yurgel *et al*., 2017 | Collinwood | 4 |
| Rhizosphere soil from roots of blueberry plants from forest, Yurgel *et al*., 2017 | Collinwood | 3 |
| Bulk soil within close proximity to roots of blueberry plants from managed field, Yurgel *et al*., 2017 | Collinwood | 4 |
| Bulk soil within close proximity to roots of blueberry plants from managed field, Yurgel *et al*., 2017 | Debert-1 | 4 |
| Rhizosphere soil from roots of blueberry plants from managed fields, Yurgel *et al*., 2017 | Collinwood | 4 |
| Rhizosphere soil from roots of blueberry plants from managed fields, Yurgel *et al*., 2017 | Debert-1 | 4 |
