# Supplementary material for: Metagenomic Functional Shifts to Plant Induced Environmental Changes
Source: Front Microbiol. 2019 Jul 26;10:1682. doi: 10.3389/fmicb.2019.01682 (PMC6676915; doi:10.3389/fmicb.2019.01682)
Supplement: Supplementary file 5 [file Table_2.docx]

Table S2. Summary of pre- and post-assembly and post-QC metagenome sequences

|  | Field bulk | Field rhizosphere | Forest bulk | Forest rhizosphere |
| --- | --- | --- | --- | --- |
| Total # of reads | 44,641,544 | 70,129,920 | 19,394,324 | 29,124,986 |
| GC content | 59.9%^A^ | 51.5%^B^ | 59.7%^A^ | 48.9%^B^ |
| Total # of reads after QC | 9,412,563 | 9,245,366 | 2,864,804 | 3,756,327 |
| GC content after QC | 59.3%^A^ | 56.9%^A^ | 59.0%^A^ | 52.2%^B^ |
| Average genome coverage | 41 | 32 | 31 | 34 |
| KOs | 4,233 | 4,434 | 3,661 | 4,372 |
| Pathways | 165 | 189 | 150 | 186 |
| Modules | 274 | 280 | 287 | 268 |

For each variable, data followed by different letters are significantly different according to Tukey’s test (P < 0.05).
