# Supplementary material for: Metagenomic Functional Shifts to Plant Induced Environmental Changes
Source: Front Microbiol. 2019 Jul 26;10:1682. doi: 10.3389/fmicb.2019.01682 (PMC6676915; doi:10.3389/fmicb.2019.01682)
Supplement: Supplementary file 10 [file Table_7.DOCX]

Table S7. KOs identified in selected KEGG pathways of xenobiotics, terpenoids and polyketides metabolism

| Pathways | % | KOs not found in the study | KOs found in bulk soil and rhizosphere metagenome |
| --- | --- | --- | --- |
| Metabolism of xenobiotics by cytochrome P450 | 36 | K13299, K00078, K07408, K07409, K07420, K07424, K13952, K00699, K00212, K07415, K07416, K07410, K07413, K07412 | K13980(**3/1**)*, K01253(**12/11**), K13951(**1/1**), K13953(**12/11**), K00001(**12/11**), K00799(**12/11**), K00121(**12/11**), K00129(**7/8**) |
| Caprolactam degradation | 79 | K01913, K07514, K07515, K00022 | K06446(**12/11**), K00055(**11/11**), K00002(**0/1**), K01825(**4/2**), K01692(**12/11**), K01053(**12/11**), K14519(**10/10**), K01782(**12**/11), K07511(**10/11**), K03379(**12/11**) |
| Geraniol degradation | 81 | K00155, K01041, K13776, | K01825(**4/2**), K11731(**11/11**), K13774(**7/10**), K01782(**11/11**), K01692(**12/11**), K13775(**12/11**), K13777(**9/11**), K13779(**4/0**), K13778(**3/6**), K01640(**12/11**), K00257(**12/11**), K00632(**12/11**), K00022(**8/10**) |
| Benzoate degradation | 76 | K07515, K07824, K01726, K04021, K07508, K07509, K00465, K07538, K01559, K07536, K14334, K04107, K04115, K04114, K07514, K05783, K07534, K07823 | K01666(**11/11**), K01031(**11/11**), K10216(**1/1**), K01032(**12/11**), K10219(**12/11**), K10218(**12/11**), K01075(**10/11**), K01607(**12/11**), K03381(**11/11**), K10217(**12/11**), K00481(**12/11**), K04099(1**2/11**), K01857(12/11), K00626(**12/11**), K05784(**2/4**), K07537(**5/3**), K07539(**1/0**), K04100(**11/11**), K04101(12/11), K00132(1/0), K00074(12/11), K04105(11/11), K04108(**3/0**), K04109(**3/1**), K01856(**9/8**), K00449(**12/11**), K00448(**11/11**), K00680(**7/10**), K14333(**12/11**), K05549(**8/9**), K05550(**5/1**), K03464(**5/9**), K02554(**10/10**), K01692(**12/11**), K01615(**0/1**), K01617(**8/9**), K00446(**12/10**), K10221(**8/5**), K10220(**12/11**), K07535(**9/7**), K00632(**12/11**), K04110(**10/11**), K04113(**0/1**), K04112(**0/1**), K04117(**6/7**), K04116(**3/4**), K01826(**10/11**), K04098(**12/11**), K01821(**12/10**), K00217(**4/4**), K01055(**12/10**), K04118(**8/9**), K00252(**12/11**), K07511(**11/11**), K07513**(8/9**), K04072(**1/1**), K04073(**10/11**) |
| Aminobenzoate degradation | 68 | K03463, K05300, K01913, K04107, K04104, K03788, K00465, K01045, K01559, K07824, K11311, K01612, K00492, K05600, K03384, K07515, K00120, K07514, K00517, K07424 | K01035(**1/7**), K01034(**6/8**), K10215(**12/11**), K01078(**10/11**), K01113(**11/10**), K10221(**9/5**), K01501(**12/11**), K01077(**7/5**), K01721(**12/11**), K03380(**9/10**), K09461(**12/11**), K01426(**12/11**), K04100(**11/11**), K04101(**12/11**), K04105(**11/11**), K04108(4/0), K04109(**3/1**), K03862(**9/10**), K03863(**6/8**), K00449(**12/11**), K00448(**11/11**), K00680(**7/10**), K01576(**12/11**), K01101(**0/2**), K01512(**12/11**), K14333(**12/11**), K01066(**11/11**), K01692(**12/11**), K05599(**1/2**), K00493(**7/10**), K04110(**11/11**), K00141(**10/10**), K01093(**0/2**), K09474(**3/1**), K14338(**2/5**), K08295(**12/11**), K01781(**10/9**), K07511(**10/11**), K04099(**12/11**) |
| Polycyclic aromatic hydrocarbon degradation | 35 | K14582, K14580, K03384, K00465, K00155, 14579, K11948, K11949, K11943, K11946, K11947, K14599, K00599, K00492, K00224, K14604, K14600, K14601, K14602, K14603, K07519, K00120, | K14581(**1/0**), K00480(**12/11**), K04102(**12/11**), K04100(**11/11**), K04101(**12/11**), K14578(**11/11**), K00449(**12/11**), K00448(**11/11**), K11944(**0/1**), K11945(**3/1**), K04099(**12/11**), K00517(**12/11**) |
| Bisphenol degradation | 42 | K05915, K05913, K00539, K01045, K00258, K01726, K00492, K00120 | K01066(**11/11**), K14520(**8/10**), K00100(**12/11**), K00517(**12/11**) |
| Xylene degradation | 85 | K10618, K10623, K10616 | K04021(**8/4**), K01666(**11/11**), K00141(**10/10**), K10217(**10/10**), K00132(**1/1**), K10619(**6/4**), K10617(**3/1**), K00055(**11/10**), K10621(**11/10**), K10622(**12/1**1), K01617(**8/**9), K02554(**10/10**), K00446(**12/10**), K10620(**0/6**), K04072(**1/1**), K10216(**1/4**), K04073(**10/11**) |
| Nitrotoluene degradation | 50 | K10679, K10678, K00195, K00194, K00197, K00196, K00193, K00192, K00198, K11180, K11181 | K00622(**0/1**), K06282(**12/11**), K06281(**12/11**), K00172(**1/1**), K00171(**3/1**), K00170(**6/3**), K03518(**12/11**), K03519(**12/11**), K00169(**6/2**), K03520(**12/11**), K10680(**12/10**) |
| Chlorocyclohexane and chlorobenzene degradation | 63 | K03384, K00539, K01860, K03268, K00224, K01856, K00492 | K01560(**12/11**), K03391(**0/1**), K01061(**12/11**), K10676(**5/9**), K00217(**4/4**), K03381(**11/11**), K00462(**1/1**), K01563(**12/11**), K00446(**12/10**), K01561(**12/11**), K04098(**12/11**), K03380(**9/10**) |
| Styrene degradation | 72 | K14481, K10566, K10438, K14482, K10437 | K01026(**11/10**), K00146(**12/11**), K10216(**1/4**), K01800(**10/11**), K01501(**12/11**), K01039(**12/11**), K01502(**9/10**), K00451(**12/11**), K01040(**12/10**), K01721(**12/11**), K01555(**12/11**), K00446(**12/10**), K01426(**12/11**) |
| Toluene degradation | 71 | K03384, K07549, K07543, K07540, K07547, K01860, K00492, K07550 | K03381(**11/11**), K03380(**9/10**), K01856(**8/8**), K07548(**4/1**), K00055(**11/10**), K07546(**6/7**), K07545(**2/1**), K07544(**5/2**), K05797(**2/2**), K00241(**12/11**), K00240(**12/11**), K00242(**12/11**), K00245(**8/6**), K00244(**9/6**), K00247(**2/3**), K00246(**3/1**), K01061(**12/11**), K00141(**10/10**), K00217(**4/4**), K00239(**12/11**) |
| Limonene and pinene degradation | 33 | K12466, K07382, K00155, K07383, K07381, K01076, K01913, K00258, K00206, K00119, K00120, K01559, K01726, K00492, | K10533(**6/6**), K01825(**4/2**), K00128(**12/11**), K00517(**12/11**), K00680(**7/10**), K01692(**12/10**), K01782(**12/11**) |
| Atrazine degradation | 47 | K14542, K01500, K06035, K08710, K03382, K05394, K01564 | K14541(**0/1**), K01428(**12/11**), K01429(**12/11**), K03383(**8/8**), K01457(**11/10**), K01430(**12/11**), K14048(**12/10**) |
| Drug metabolism - other enzymes | 72 | K01044, K03927, K07424, K01431, K00699, K07411 | K00857(**12/9**), K00088(**12/10**), K00760(**12/11**), K00622(**0/1**), K00876(**9/8**), K01951(**12/11**), K00207(**7/2**), K00569(**3/2**), K01519(**0/3**), K00106(**0/2**), K00758(**11/11**), K00757(**4/2**), K01195(**12/10**), K01464(**12/11**), K01489(**11/10**), K13421(**1/0**) |
| Dioxin degradation | 71 | K04021, K08690, K10222, | K01666(**11/11**), K00132(**1/0**), K08689(**1/0**), K02554(**10/10**), K01821(**12/10**), K01617(**8/9**), K00462(**1/1**), K00480(**12/11**), K04072(**1/1**), K04073(**9/10**) |
| Fluorobenzoate degradation | 62 | K03384, K01860, K14417, K14418, K08686, | K01061(**12/11**), K05783(**4/5**), K00217(**4/4**), K01721(**12/11**), K05550(**5/3**), K03391(**0/1**), K03381(**11/11**), K05549(**8/8**), K01856(**8/8**), K05784(**2/4**) |
| Naphthalene degradation | 39 | K14586, K14585, K14584, K14583, K14582, K14580, K03384, K01726, K00155, K14579, K00465, K01041, K01670, K05915, K00492, K00120, K00516 | K14581(**1/0**), K00480(**12/11**), K00152(**0/5**), K14578(**11/11**), K00680(**7/10**), K13953(**12/11**), K13954(**4/0**), K00001(**12/11**), K00121(**12/11**), K04072(**1/1**), K00257(**12/11**) |
| Chloroalkane and chloroalkene degradation | 58 | K14420, K02591, K14029, K14419, K14422, K00120, K14421, K00531, K00100, K00539, K01564 | K00128(**12/11**), K14028(**10/9**), K00114(**12/11**), K13953(**12/11**), K13954(**4/0**), K08726(**0/1**), K00001(**12/11**), K00148(**12/11**), K02588(**1/1**), K01563(**12/11**), K00121(**12/11**), K01561(**12/11**), K01560(**12/11**), K04072(**1/1**), K02586(**1/1**) |
| Ethylbenzene degradation | 72 | K14579, K10700, K14580, | K01666(**11/11**), K10701(**6/3**), K14578(**11/11**), K10702(**3/3**), K14581(**1/0**), K02554(**10/10**), K00680(**7/10**), K00632(**12/11**) |

* - Found in bulk/found in rhizosphere.
